# MiR-99a Antitumor Activity in Human Breast Cancer Cells through Targeting of mTOR Expression

**DOI:** 10.1371/journal.pone.0092099

**Published:** 2014-03-17

**Authors:** Yu Hu, Qin Zhu, Lili Tang

**Affiliations:** 1 Department of Breast Surgery, Xiangya Hospital, Central South University, Changsha, China; 2 Department of General Surgery, Xiangya Hospital, Central South University, Changsha, China; Columbia University, United States of America

## Abstract

MicroRNAs (miRNAs) play an important role in human tumorigenesis as oncogenes or tumor suppressors. miR-99a has been reported as a tumor suppressor gene in various cancers in humans. However, only limited information about the function of miR-99a in human breast cancers is available. Here we investigated the expression of miR-99a in breast cancer tissue specimens and its antitumor activity in breast cancer cells. We initially identified that the expression of miR-99a was significantly reduced in four breast cancer cell lines. More importantly, we found downregulation of miR-99a in breast cancer specimens from ten different patients. We then analyzed the mechanism of miR-99a in inhibiting tumorigenesis. Cell-based assays that showed overexpression of miR-99a not only reduced breast cancer cell viability by inducing accumulation of cells at sub-G1 phase and cell apoptosis, but also inhibited tumorigenicity in vivo. As a critical miR-99a target, we have shown that the function of mammalian target of rapamycin (mTOR) was greatly inhibited by miR-99a-based Luciferase report assay; overexpression of miR-99a reduced the expression of mTOR and its downstream phosphorylated proteins (p-4E-BP1 and p-S6K1). Similar to restoring miR-99a expression, mTOR downregulation suppressed cell viability and increased cell apoptosis, whereas restoration of mTOR expression significantly reversed the inhibitory effects of miR-99a on the mTOR/p-4E-BP1/p-S6K1 signal pathway and the miR-99a antitumor activity. In clinical specimens and cell lines, mTOR was commonly overexpressed and its protein levels were statistically inversely correlated with miR-99a expression. Taken together, these results have demonstrated that miR-99a antitumor activity is achieved by targeting the mTOR/p-4E-BP1/p-S6K1 pathway in human breast cancer cells. This study suggests a potential therapeutic strategy to effectively control breast cancer development.

## Introduction

MicroRNAs (miRNAs) are a group of small (18-25-nucleotide long), endogenous non-coding RNA molecules. These miRNAs can regulate gene expression post-transcriptionally through binding to the 3′-untranslated region (3′-UTR) of target genes to promote mRNA degradation or protein translation inhibition[1]. Thus, they play important roles in various biological processes, such as embryo development, cell proliferation and differentiation, and carcinogenesis[1]–[4]. A great number of studies have demonstrated that miRNAs function as onco- or tumor suppressor genes and that their aberrant expression contributes to human diseases such as cancer[2]–[4]. To date, extensive studies have reported aberrant expression of miRNAs such as miR-122, miR-200c, and miR-10b in breast cancer[5]–[7]. Further investigation of miRNA involvement in breast cancer could help us better understand the molecular mechanisms responsible for breast cancer development and lead to novel strategies for effective control of breast cancer.

The tumor suppressor gene miR-99a is frequently lost or expressed at reduced levels in various human cancers. For example, miR-99a was found to be down regulated in esophageal squamous cell carcinoma tissues and reduced miR-99a expression was correlated with worse overall patient survival. Overexpression of miR-99a by transient gene transfection inhibited esophageal cancer cell proliferation and induced apoptosis[8]. miR-99a was also found to induce cell cycle arrest at G1 phase and suppress tumorigenicity in renal cell carcinoma[9]. Both miR-99a and the related miR-99b can modulate TGF-beta-induced epithelial to mesenchymal transition in normal murine mammary gland cells[10]. Moreover, induction of cell cycle arrest by miR-99a may suppress expression of insulin-like growth factor 1 receptor (IGF-1R) and mammalian target of rapamycin (mTOR) in hepatocellular carcinoma cells[11]. Expression of miR-99a inhibits the growth of prostate cancer cells and reduces the expression of prostate-specific antigen by targeting chromatin-remodeling factors such as SMARCA5, SMARCD1 and the growth regulator kinase mTOR in vivo[12]. miR-99a expression also reduces cell proliferation and induces cell apoptosis by targeting estrogen receptor 1(ESR1) in endometrial cancer[13]and IGF-1R in head and neck squamous cell carcinoma cells[14]. Taken altogether, these studies demonstrate miR-99a antitumor activity in different human cancers. However, to date, there has been no study reporting the role of miR-99a in human breast cancer. Thus, our study investigated the biological functions and mechanisms of miR-99a as antitumor miRNA by repressing the activity of mTOR in breast cancer cells in vitro as well as in nude mouse xenografts. This study has further characterized that miR-99a is a tumor suppressor by directly targeting mTOR in human breast cancers.

## Materials and Methods

### Clinical breast cancer samples

Ten surgical specimens (both tumor and adjacent normal tissue) were obtained from patients in Xiangya Hospital (Central South University, Changsha, China). Written informed consent was obtained from each patient and this study was approved by the Human Research Ethics Committee of the Xiangya Hospital. The histological diagnosis was verified by an experienced pathologist. Tissue samples were immediately frozen in liquid nitrogen and stored at −80°C until used. No patient had received chemotherapy or radiation therapy treatment before surgery. The clinical stage was defined according to TNM staging system.

### Cell lines, cell culture, and miRNA transfection

Human normal breast cell lines HBL-100, human breast cancer cell lines SK-BR-3 and MDA-MB-435s were obtained from Institute of Biochemistry and Cell Biology (Shanghai,China). Human breast cancer cell lines MCF-7 and MDA-MB-231 were obtained from the Cell Center at the Xiangya School of Medicine (Changsha, China). All cell lines were grown in RPMI-1640 medium supplemented with 10% fetal bovine serum (FBS, Gibco Life Technologies, USA), 100 IU/ml penicillin, and 100 μg/ml streptomycin at 37°C in a humidified incubator with 5% CO_2_.

miR-99a mimics, mTOR siRNA and scrambled negative control (NC) were chemically synthesized by GenePharma (Shanghai, China). Breast cancer (BC) cells (MCF-7 and MDA-MB-231) were plated in 6-well plates at 50% confluence the day before transfection. The next day, 50 nM of mTOR siRNA or miR-99a mimics was transfected into BC cells using Lipofectamine 2000 (Invitrogen, Carlsbad, USA) according to the manufacturer's protocol. Total RNAs or proteins were prepared at 72 h post-transfection and used for quantitative reverse transcription (qRT)-PCR or western blot analysis respectively. For the mTOR cDNA plasmid without 3′-UTR (Addgene, Cambridge, USA) and miR-99a mimics combination experiment, MCF-7 cells were first transfected with miR-99a mimics (50 nM). 48 h later, these cells were co-transfected with mTOR cDNA plasmid (2 μg) and miR-99a mimics (50 nM) for another 72 h. Cells were used for apoptosis assays or western blot analyses. miR-99a expression plasmid CMV-miR-99a and negative control CMV-NC (nontargeting control sequence) plasmid were purchased from GenePharma (Shanghai, China). Cells were transfected with CMV-miR-99a (2 μg) or CMV-NC (2 μg) using Lipofectamine 2000 (Invitrogen) according to manufacturer's instructions. For the in vivo tumorigenicity assay, we generated a stable cell line of MDA-MB-231 transfected by either CMV-miR-99a or CMV-NC. The stable cell lines were screened by incubation with Blasticidin (BioVision, Milpitas, USA) and maintained in medium containing 10 μg/mL Blasticidin. After a 4-week selection period, MDA-MB-231 cell lines with stable expression of miR-99a or control were obtained; these were designated MDA-MB-231-99a and MDA-MB-231-NC, respectively.

### RNA isolation and qRT-PCR analyses

Total cellular RNA was isolated from breast cancer cells using TRIzol reagent (Invitrogen) according to the manufacturer's protocol. Expression of miR-99a was detected with the All-in-One™ miRNA qRT-PCR Detection Kit (GeneCopoeia, Rockville, MD), and normalized to U6 snRNA using the 2^(−ΔΔCt)^ method. mTOR mRNA expression was quantified using the SYBR PrimeScript RT-PCR Kit II (Takara, Shiga, Japan) and normalized by GAPDH using the 2^(−ΔΔCt)^ method. The PCR primers were as follows: miR-99a, forward, 5′-AAC CCG TAG ATC CGA TCT TGT G-3′ and reverse, 5 ′-TGG TGT CGT GGA GTC G-3′; U6, forward, 5′-GCT TCG GCA GCA CAT ATA CTA AAA T-3′ and reverse, 5 ′-CGC TTC ACG AAT TTG CGT GTC AT-3′; mTOR, forward, 5′-ACA GCC CAG GCC GCA TTG TC-3′ and reverse, 5′-TCC AGG GAC TCC GTC AGG CG-3′; GAPDH, forward, 5′-CAA TGA CCC CTT CAT TGA CC-3′ and reverse, 5′-GAC AAG CTT CCC GTT CTC AG-3′. All qRT-PCR amplifications were performed in triplicate.

### Protein extraction and western blot

Cells and tissue powder were lysed in a RIPA lysis buffer (Beyotime, Beijing, China) and protein concentration was measured by a BCA protein assay kit (Thermo Scientific, Pittsburgh PA,USA). After that, these protein samples were separated on 10% SDS-PAGE and then transferred onto PVDF membranes (Millipore, Massachusetts, USA). Western blot analysis was performed as described previously[11]with primary antibodies against mTOR (Cat. #4517, Cell Signaling Technology, Danvers, USA), phosphorylated 4E-BP1 at T^37^/T^46^ (Cat. #2855, Cell Signaling Technology,), phosphorylated S6K1 at T^389^ (Cat. ab129230, Abcam, Cambridge, USA), 4E-BP1 (Cat. Ab32130, Abcam), S6K1 (Cat. #2708, Cell Signaling Technology) and GAPDH (G9545, Sigma-Aldrich, St. Louis, USA). Positive protein signals were detected with an enhanced chemiluminescence (ECL) detection kit (GE Healthcare, Waukesha, USA). The images were quantified by densitometry (Beckman, South Pasadena, Canada).

### Luciferase reporter assay

The wild-type 3′-UTR fragment of human mTOR mRNA containing the putative miR-99a binding site was amplified by PCR and cloned into the XbaI site of the pGL3-control vector (Promega, USA) which is the downstream of the luciferase reporter gene. The corresponding mutant constructs were created by mutating the seed regions of the miR-99a-binding sites (5′-UACGGGU -3′ to 5′-AUGCCCA -3′). The nucleotide sequences of primers for mTOR-3′UTR clone were: 5′- CGGGGTACCAGATGTGCCCATCACGTTTT-3′ (forward) and 5′- CCGGAATTCTGGTGTCTAGACATGGCTACACTT-3′ (reverse) and the mutagenesis primers were: 5′- CCATAACTTTAGAAAGCTACACTTTGACTTAACTCAC -3′ (forward) and 5′- GTGAGTTAAGTCAAAGTGTAGCTTTCTAAAGTTATGG -3′ (reverse). For the luciferase reporter assay, the 293T cell line was co-transfected with luciferase reporter vectors and miR-99a mimics using Lipofectamine 2000. A 1.0 ng pRL-TK Renilla Luciferase construct was used for normalization. After 48 h, the luciferase activity was analyzed by the Dual-Luciferase Reporter Assay System according to the manufacture's protocols (Promega, Madison, USA).

### Cell viability assay

Cell viability capacity was assessed by detection of formazan production from the CellTiter 96 AQueous One Solution Cell Proliferation Assay kit (Promega, Madison, WI, USA). Briefly, cells were plated in 96-well plates at a density of 1×10^4^ cells/well and then transfected with miR-99a mimics, mTOR siRNA, or scrambled negative control (NC) using Lipofectamine 2000 (Invitrogen) according to manufacturer's instructions. The colorimetric MTT assay was performed to measure the absorbance rate of cells and the samples were detected at 0, 12 h, 24 h, 48 h and 72 h after gene transfection. This assay was performed in triplicate and repeated thrice.

### Flow cytometry analysis of apoptosis and cell cycle

After transfected with miR-99a mimics, mTOR siRNA, or negative control for 72 h, the cells were incubated in serum-free medium for 24 h for cell synchronization. For apoptosis analysis, cultured cells were harvested by trypsinization and washed twice by PBS. Cells (1×10^6^ cells/mL) from each sample were processed with the Annexin V FITC/PI apoptosis detection kit (BD Biosciences) according to the manufacturer's instructions. For cell cycle analysis, the cells were typsinized, washed twicewith PBS, and then fixed with 75% ethanol on ice for 2 h. The fixed cells were spun down and resuspended in 600 μl PBS containing 0.1% RNase (Sigma) for RNA digestion at 37°C for 1 h. Finally, the cells were stained with PI for 15 min in the dark room before being measured by flow cytometry. A FACS Calibur instrument (BD Biosciences) was used for this assay. The experiments were performed in triplicate and repeated thrice.

### In vivo tumor xenograft model

To assess the inhibitory effect of miR-99a in breast cancer cells, we performed the in vivo tumor xenograft assay using 18 female, 4 to 5-weekold BALB/c nude mice. In this experiment, we followed the Shanghai Medical Experimental Animal Care Commission and the local ethics guidelines for animal research. The mice, which were divided into three treatment groups with 6 mice in each group, i.e. MDA-MB-231 cells with CMV-miR-99a, CMV-NC, and MOCK groups (1×10^6^), were subcutaneously injected in the left flank to establish the tumor xenograft. Tumor size was measured for length (L) and width (W) every 5 days for 30 days, and volume was calculated using the formula (L×W^2^)×0.5. All animal procedures were approved by the Animal Ethics Committee of the Xiangya Hospital.

### Statistical analysis

Each experiment was repeated at least three times. Quantitative data were summarized as mean ± SD. The differences and correlations between two groups were assessed by the Mann-Whitney U test or Pearson's correlation test. Multiple group comparison was analyzed by using ANOVA with a post-test for subsequent individual group comparisons. All statistical analyses were performed using SPSS 13.0 (SPSS, Chicago, IL). Data were considered to be statistically significant when p<0.05.

## Results

### The miR-99a expression is down-regulated in both human breast cancer tissues and cell lines

**Figure 1:**
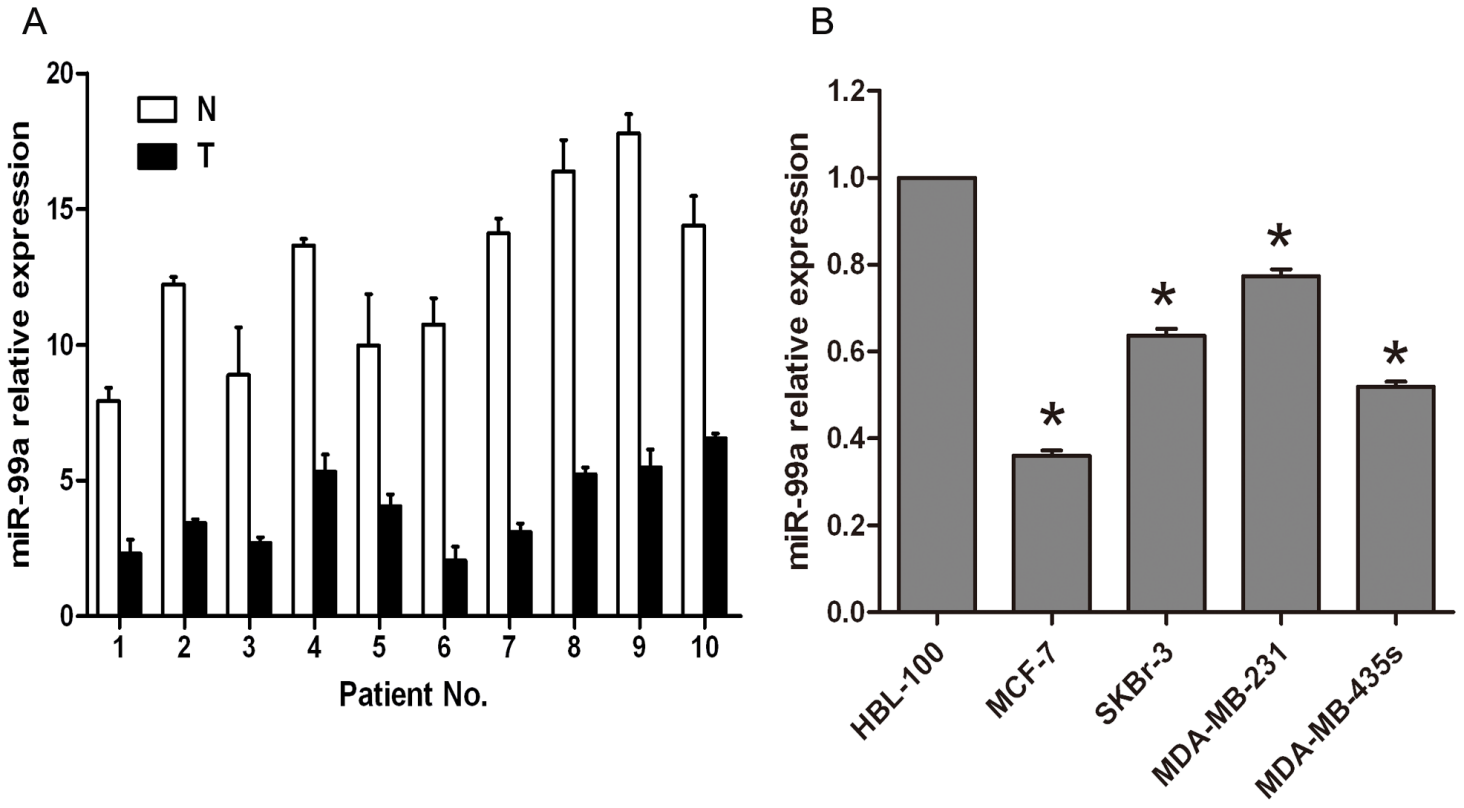
Differential expression of miR-99a in human breast cancer tissues and cell lines. (A) qRT-PCR analysis of miR-99a expression in 10 human breast cancer tissues vs. the distant non-tumor tissues. (B) qRT-PCR analysis of miR-99a expression in four human breast cancer cell lines and a normal human breast epithelial cell line. The level of miR-99a expression was normalized to U6 and compared to the normal cells. Data were normalized to U6 snRNA and represent the mean ± SD of three independent duplicate experiments. *, P<0.05 significant differences from HBL-100.

Previous studies have shown that miR-99a was down regulated in several human cancers including esophageal squamous cell carcinoma, hepatocellular carcinoma cells and prostate cancer cells[8], [11], [12], but most studies were performed on different cancer cell lines and no study was conducted directly from patients in clinic. In this study, we first assessed miR-99a expression in breast cancer tissue from patients and then from breast cancer cell lines. Analysis of qRT-PCR showed that miR-99a expression was reduced in all 10 cases of human breast cancer tissues compared to the normal tissue counterparts (Figure 1A). Moreover, we also detected miR-99a expression in four breast cancer cell lines (i.e., MCF-7, MDA-MB-231, MDA-MB-435s, and SKBR-3) and a normal human breast epithelial cell line HBL-100. As shown in Figure 1B, miR-99a expression was significantly reduced in human breast cancer cell lines compared to HBL-100 cells. The data have shown that the reduction of miR-99a was found in both breast cancer tissues from patients and human breast cancer cell lines, indicating that downregulation of miR-99a is clearly involved in human breast cancer development.

### Overexpression of miR-99a inhibits breast cancer cell viability and induces apoptosis

**Figure 2:**
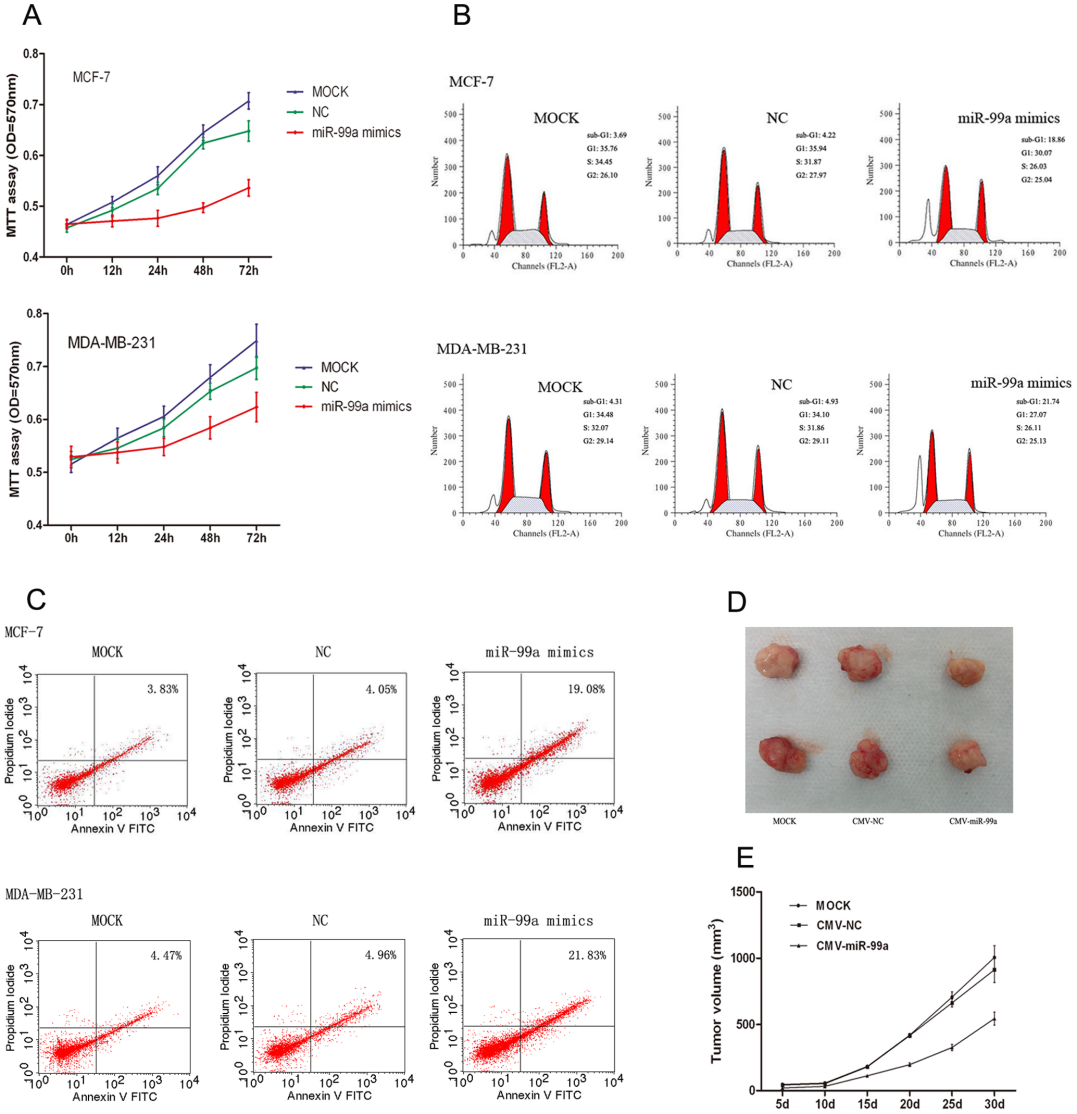
The inhibitory effect of miR-99a mimics on human breast cancer cells. (A) Cell viability MTT assay. The cell viability curve was constructed consecutively for up to 72 h after transfection of MCF-7 and MDA-MB-231 cells with Lipofectamine 2000 only (MOCK), scrambled negative control (NC) and miR-99a mimics. (B) Flow cytometry analysis of cell cycle. At 72 h post-transfection, MCF-7 and MDA-MB-231 cell lines treated with miR-99a mimics showed a remarkable increased sub-G1 phase population compared with MOCK and NC groups. (C) Flow cytometry analysis of apoptosis. After 72 h treatment, transfection of miR-99a mimics into MCF-7 and MDA-MB-231 cell lines resulted in a significant increase percentage of apoptotic cells compared with the control. (D) The tumors shown are from the final time point (30_day). Xenograft sizes from six representative nude mice from three treatment groups (MDA-MB-231 cells with CMV-miR-99a, CMV-NC and MOCK). (E) The xenograft growth analysis after breast cancer cell inoculation. The average size of the tumors was measured every 5 days and shown in the curves (P<0.05 compared to the control cells).

To determine whether miR-99a is involved in tumor suppression in human breast cancer cells, we assessed the effects of miR-99a's cell viability in breast cancer cells by transfecting with Lipofectamine 2000 only (MOCK), negative control (NC), or miR-99a mimics into MCF-7 and MDA-MB-231 cells. We showed that (i) overexpression of miR-99a led to a notable reduction of cell viability in both MCF-7 and MDA-MB-231 cells compared to control cells (Figure 2A; p<0.05); (ii) transfection of miR-99a mimics resulted in remarkable accumulation of cell cycle at sub-G1 phase compared to the control groups (p<0.05); the accumulation of sub-G1 cell proportion indicates cells undergoing apoptosis[15], [16] (Figure 2B; p<0.05) (iii) the effect of miR-99a mimics on inducing cell apoptosis was significantly increased compared to the control groups, (Figure 2C; p<0.05). Together, these results indicate that miR-99a overexpression clearly inhibits the proliferation of human breast cancer cells.

### Overexpression of miR-99a inhibits breast cancer cell xenograft formation

To further confirm the in vitro results above, we performed a nude mouse xenograft assay by injecting MDA-MB-231 cells stably transfected with CMV-miR-99a into nude mice. We found that tumor growth in the CMV-miR-99a group was significantly slower than that of the control groups (Figure 2D). The average tumor volume of the CMV-NC group was 1.68 fold higher than that of the CMV-miR-99a group after 30 days post-inoculation (Figure 2E).

### MTOR is a direct target of miR-99a in breast cancer cells

**Figure 3:**
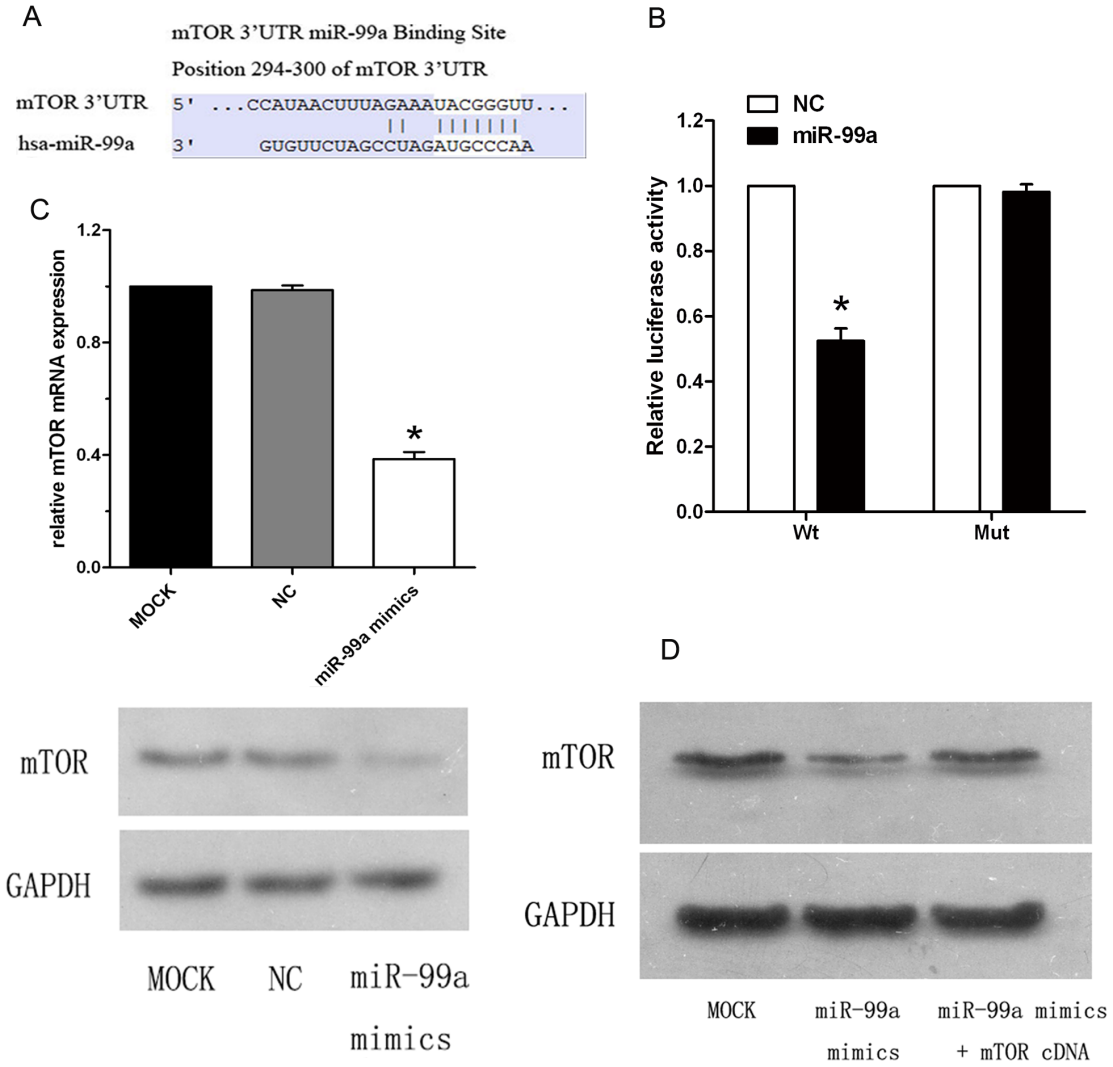
mTOR is a direct target of miR-99a in MCF-7 breast cancer cell line. (A) Targetscan prediction of miR-99a targeting of mTOR at the mTOR mRNA 3′-UTR.(B) miR-99a-mediated directly inhibitory effect of mTOR by luciferase reporter assays in which the expression of the reporter containing mTOR 3′UTR was suppressed by miR-99a, but not in the mutated construct. (C) The miR-99a-mediated suppression of mTOR at both mRNA and protein levels in MCF-7 cells and analyzed by qRT-PCR and Western blot, respectively (P<0.05 compared to control cells). (D) mTOR rescue analysis. The miR-99a-mediated suppression of mTOR in MCF-7 cells was rescued by transfection of mTOR cDNA plasmid without the 3′-UTR.

To identify miR-99a target genes, we searched the Targetscan and Pictar databases and found that mTOR was one of the predicted miR-99a targets whose 3′-UTR contained putative miR-99a target sites (Figure 3A). Indeed, several groups have reported this result in different human cancers[8], [11], [12], [17]. To confirm that mTOR is a direct target of miR-99a, we cloned the 3′UTR of mTOR into a reporter plasmid downstream from luciferase and performed reporter assays. The result showed that the relative luciferase activity of the reporter which contained wild-type 3′UTR of mTOR was notably decreased in miR-99a group compared to NC group. In contrast, the relative luciferase activity of the mutant mTOR 3′UTR reporter had no significant difference from the control group and failed to respond to miR-99a (Figure 3B). To further determine whether miR-99a inhibits mTOR expression in human breast cancer cells, we transfected miR-99a mimics into MCF-7 cells and analyzed mRNA and protein levels by qRT-PCR and immunoblot. In MCF-7 cells transfected by miR-99a mimics, mTOR was significantly decreased at the levels of both mRNA (0.39 fold) and protein (0.37 fold) compared to control cells (Figure 3C), consistent with previous studies[11], [12], [17]. However, the MCF-7 cells transfected by mTOR cDNA plasmid that lacks 3′-UTR dramatically overcame the repressive effect of miR-99a mimics (Figure 3D). This indicates that miR-99a directly targets the mTOR through its 3′-UTR and, in turn, inhibits its expression.

### Down-regulation of miR-99a highly correlates with up-regulation of mTOR expression in both human breast cancer tissues and cell lines

**Figure 4:**
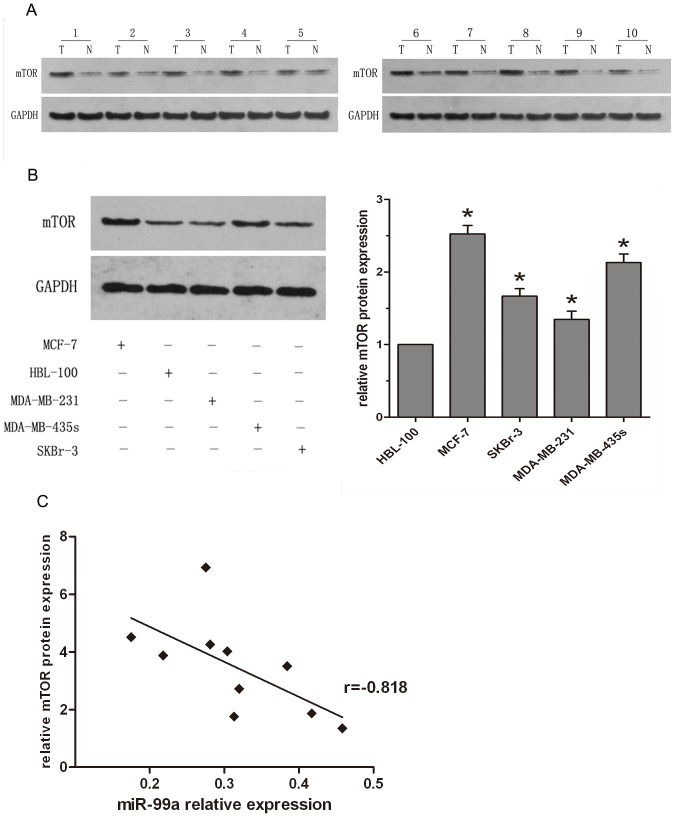
Statistical correlation between the expression levels of miR-99a and mTOR protein in breast cancer tissues and cell lines (A) mTOR protein was analyzed by Western blotting in 10 pairs of breast cancer tissues. (B) mTOR protein was analyzed in four different breast cancer cell lines and a breast epithelial cell line. Data were normalized to GAPDH protein and represent the mean ± SD of three independent duplicate experiments. *, P<0.05 significant differences from HBL-100. (C) The inverse correlation in breast cancer tissues was analyzed by Pearson's correlation method.

To further confirm that miR-99a inhibits mTOR expression in breast cancer, we analyzed the protein levels of mTOR from ten pairs of breast cancer and normal tissue specimens by immunoblot. Compared to the non-tumor counterparts, the expression of mTOR protein was significantly increased in tumor tissues from ten different patients (Figure 4A). The same is also true for breast cancer cell lines. i.e., mTOR expression in these four breast cancer cell lines were markedly higher than that in breast epithelial cell line, and was associated with reduced miR-99a expression (Figure 4B). We then compared mTOR and miR-99a expression in these tissues and found that among these 10 pairs of breast cancer tissues, there was a statistically significant inverse correlation between mTOR and miR-99a expression (Figure 4C).

### MiR-99a-mediated inhibition of mTOR is involved in tumor suppression of breast cancer cells

**Figure 5:**
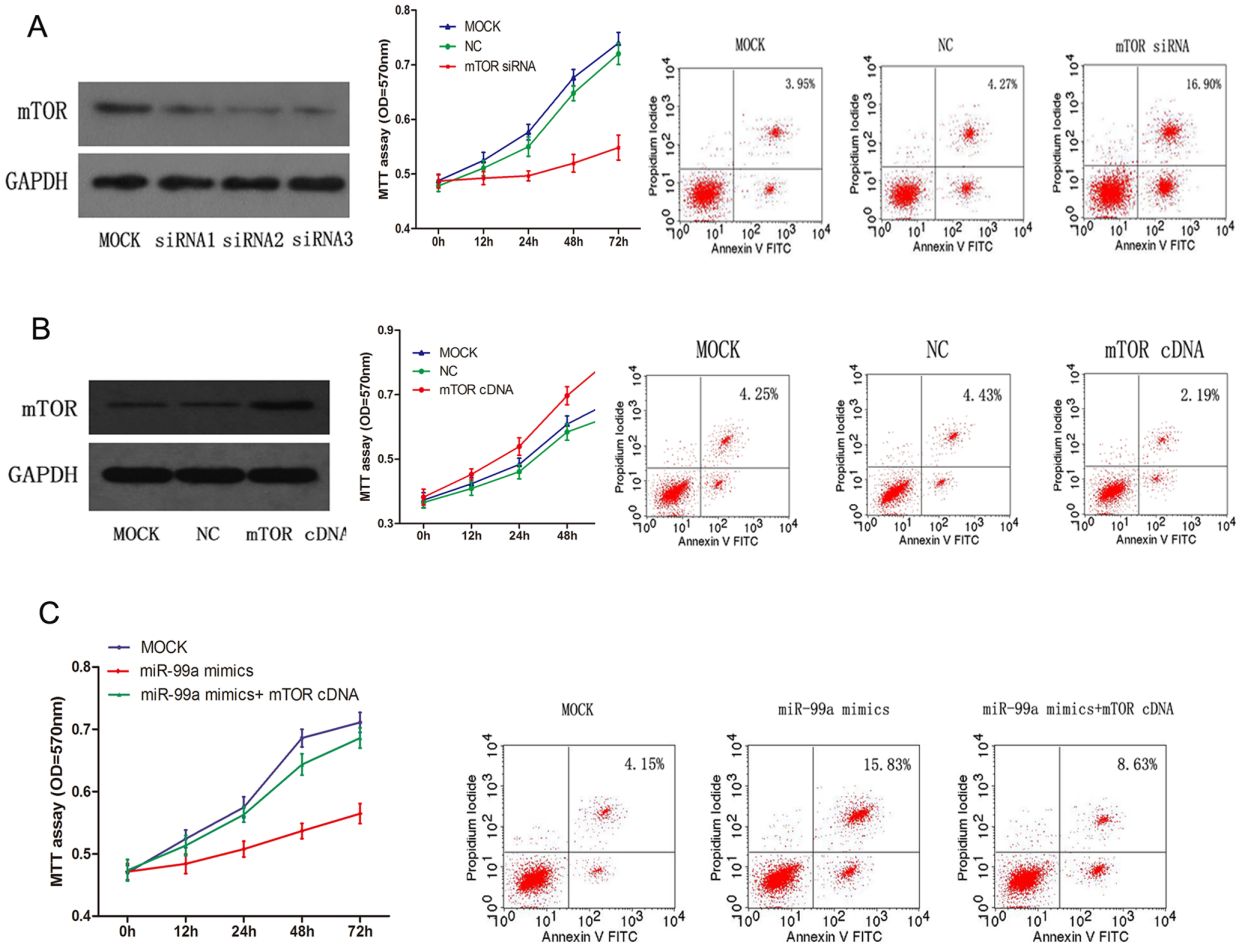
mTOR mediated miR-99a antitumor activity in breast cancer cells. (A) mTOR depletion-mediated cell viability and apoptosis. MCF-7 cells were transfected with MOCK, NC and mTOR siRNA respectively. An MTT cell viability assay was performed at 0, 12, 24, 48 and 72 h. Cell apoptosis was analyzed at 72 h post-transfection, as described in Materials and methods. (B) Cell survival analysis by overexpression of mTOR in breast cancer cells. MDA-MB-231 cells were transfected with MOCK, NC and mTOR cDNA plasmid without the 3′-UTR respectively. (C) miR-99a-mediated cell survival and mTOR rescue analysis in breast cancer cells. MCF-7 cells were co-transfected by miR-99a mimics and mTOR cDNA plasmid without the 3′-UTR followed by MTT assay and apoptosis analysis, as described in Materials and methods.

To further test whether miR-99a-mediated mTOR inhibition confers antitumor activity in breast cancer cells, we first confirmed the biological functions of miR-99a mimics-transfected breast cancer by inhibiting mTOR expression. We inhibited endogenous mTOR expression to assess whether it can mimic the biological effect of miR-99a. Indeed, knockdown of mTOR expression by mTOR-siRNA caused a significant inhibition of breast cancer cell viability and a notable increase in cell apoptosis vs. the control group, in a manner similar to those transfected with miR-99a mimics (Figure 5A). Ectopic expression of mTOR by mTOR cDNA plasmid without the 3′-UTR had the contrary effect (Figure 5B). Next, we co-transfected miR-99a mimics and mTOR cDNA plasmid that lacks the 3′-UTR into breast cancer cells to determine whether the ectopic expression of mTOR could reverse the inhibitory effect of miR-99a on breast cancer cells. The results showed that the inhibition of breast cancer cell proliferation and the acceleration of apoptosis by miR-99a mimics were rescued by restoration of mTOR expression (Figure 5C).

### The levels of p-4E-BP1 and p-S6K1, two downstream molecules of mTOR signaling pathway, are significantly reduced upon overexpression of miR-99a in human breast cancer cells

**Figure 6:**
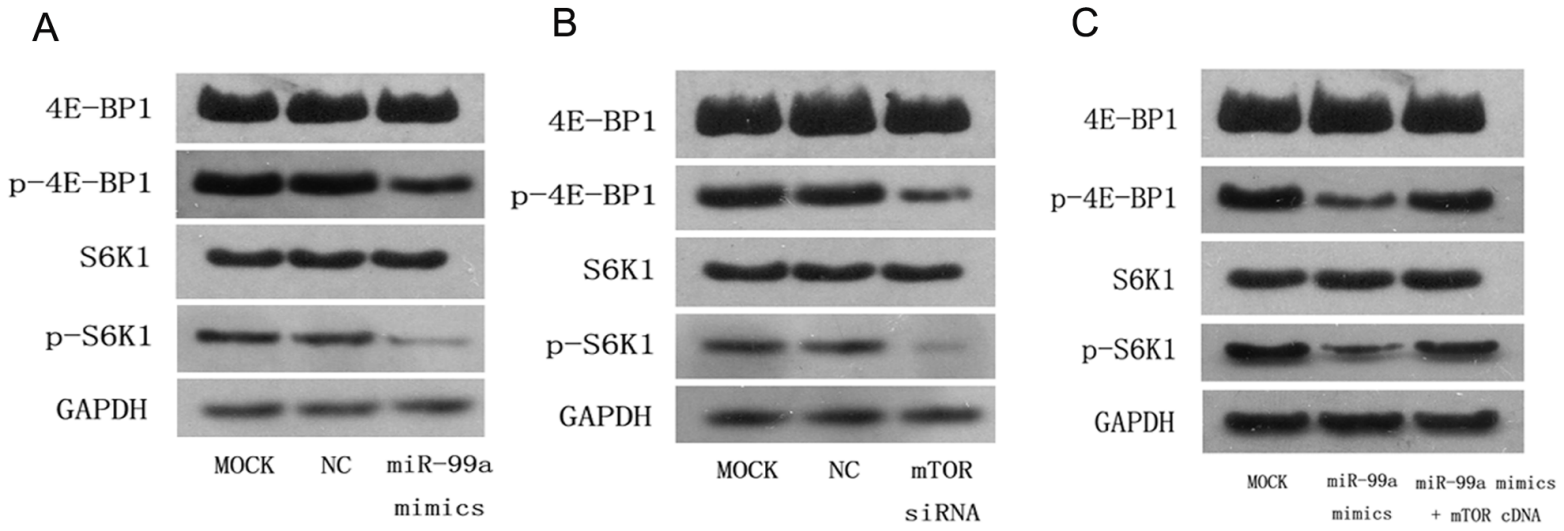
miR-99a regulated expression of mTOR downstream signaling pathway genes p-4E-BP1 and p-S6K1. (A-C) Immunoblot analyses of total and phosphorylated 4E-BP1 and S6K1 from MCF-7 breast cancer cells transfected with miR-99a mimics (A), mTOR siRNA(B), and co-transfection of both miR-99a mimics and mTOR cDNA plasmid without the 3′-UTR(C). Levels of p-4E-BP1 and p-S6K1 proteins were both markedly decreased after miR-99a mimics transfection in MCF-7 cells, Knockdown of mTOR expression using mTOR siRNA also distinctly reduced levels of p-4E-BP1 and p-S6K1 proteins, however, total 4E-BP1 and S6K1 protein showed no change; The inhibitory effects of miR-99a mimics on p-4E-BP1 and p-S6K1 could be negated by re-expression of mTOR.

Since 4E-BP1 and S6K1 are two direct downstream molecules of mTOR signaling pathway, we further investigated the expression of total and phosphorylated 4E-BP1 and S6K1 in MCF-7 cells following miR-99a overexpression or mTOR inhibition. We found that the endogenous levels of both p-4E-BP1 and p-S6K1 proteins were markedly decreased after miR-99a mimics transfection compared to the control cells (Figure 6A), and inhibition of mTOR expression using mTOR siRNA also distinctly reduced levels of p-4E-BP1 and p-S6K1 proteins (Figure 6B). Interestingly, however, total 4E-BP1 and S6K1 protein remained the same. Furthermore, re-expression of mTOR in MCF-7 cells could significantly overcome the inhibitory effects of miR-99a mimics on regulation of mTOR/p-4E-BP1/p-S6K1 signal pathway (Figure 6C). Together, the data suggests an important role of mTOR as a mediator of the biological effects of miR-99a.

## Discussion

Although miR-99a is a well-studied tumor suppressor in a variety of human cancers, its function in human breast cancers is not very clear. Thus, in the current study, we have assessed the role of miR-99a in breast cancer cells, and then explored the underlying molecular mechanism responsible for miR-99a antitumor activity. We have established that (i) miR-99a expression was significantly reduced in breast cancer tissues and cells lines; (ii), Ectopic expression of miR-99a greatly decreased breast cancer cell viability, induced apoptosis and inhibited tumorigenicity in nude mice; (iii), consistent with previous studies, mTOR was further confirmed as the direct target of miR-99a. MiR-99a greatly reduced the expression of mTOR on both mRNA and protein levels, and inhibited the phosphorylated form of the mTOR downstream targets (p-4E-BP1 and p-S6K1), re-expression of mTOR protein overcame the inhibitory effect of miR-99a mimics on the mTOR/p-4E-BP1/p-S6K1 signal pathway and miR-99a antitumor activity. Furthermore, miR-99a expression was statistically inversely correlated with mTOR expression in both breast cancer tissue specimens and cell lines. Our current data demonstrate that reduced miR-99a expression in breast cancer tissues and cells result in mTOR overexpression, which in turn contributes to breast cancer development. However, the open question is that the mechanism of deregulated miR-99a in a variety of human cancers has not been defined in the field based on previous studies as well as ours. Future studies will also investigate whether the miR-99a-mediated inhibition of mTOR signaling pathway could be used as a potential therapeutic strategy to effectively control breast cancer in clinic.

Several studies have shown reduced or lost miR-99a expression in different human cancers such as ovarian, lung, head and neck and colon cancer[18]–[21], in which miR-99a has been identified as a tumor suppressor, despite one study reported that miR-99a was overexpressed in gastric cancer as an oncogene[22]. In order to resolve this controversial result, our study has further confirmed that miR-99a is tumor suppressor by its dramatic down-regulation in breast cancer tissue samples and cell lines compared to the normal breast tissues. Mueller et al showed that expression of the miR-99 family was upregulated in response to radiotherapy of breast cancer cells and reduced tumor cells' ability to repair damaged DNA[23]. Turcatel et al used mouse mammary gland cells to demonstrate that miR-99a can suppress a complex network of transforming breast epithelial to mesenchymal cells and proliferation and migration of breast cancer cells[10]. However, the precise mechanism of miR-99a antitumor activity remains to be defined. Our current study has identified several biological functions of miR-99a including reduction miR-99a expression in breast cancer tissues and cell lines, reduction of breast cancer cell viability and induction of apoptosis and inhibition of tumorigenesis in vivo after overexpression of miR-99a, as well as molecular mechanism of miR-99a-mediated inhibitory effect of mTOR in human breast cancer cells.

Previous studies have reported that miR-99a can inhibit mTOR expression by directly targeting its 3′-UTR in numerous human cancers including prostate cancer cells, childhood adrenocortical tumor cells, and c-Src-transformed cells[12], [17], [24]. We have extended these studies and shown that the mTOR mRNA 3′-UTR contains putative miR-99a target sites[11], [12], [17]. Our results have established that mTOR was a direct target of miR-99a in breast cancer cells. We first showed that expression of miR-99a was able to inhibit expression of mTOR mRNA and protein in MCF-7 cells. Our ex vivo data also showed a statistically significant inverse correlation between expression of mTOR and miR-99a in breast cancer tissues and cell lines. Moreover, the repression of mTOR expression by miR-99a was rescued by transfection of mTOR cDNA plasmid that lacks the 3′-UTR. This findings support a model where miR-99a directly inhibits mTOR expression in breast cancer via binding to mTOR 3′-UTR.

mTOR is a protein kinase in the PI3K/Akt signaling pathway and mTOR protein can phosphorylate and activate its downstream effectors S6K1 and 4E-BP1 in control of protein translation (such as CDK and cyclin D1) and regulate cell proliferation and cell cycle[25], [26]. Aberrant gene expression of mTOR pathway alters cell growth and apoptosis in many cancer types such as prostate cancer, lung cancer, acute myelogenous leukemia, hepatocellular carcinoma, gastric cancer[27]–[31] and breast cancer. In breast cancer, 44.9% of tumor tissues had increased levels of mTOR, while 71.9% of invasive breast cancer tissues expressed high level of phosphorylated S6K1 protein[32]. Other studies reported overexpression of mTOR and its substrate in breast cancer tissue and cell lines[33], [34]. In our study, we found that knockdown of mTOR expression using mTOR siRNA decreased breast cancer cell viability and induced apoptosis, a similar outcome to that of tumor cells transfected with miR-99a mimics. Furthermore, the inhibition of breast cancer cell viability and the acceleration of apoptosis by miR-99a mimics were rescued by restoration of mTOR expression. The results indicated that mTOR was required for the miR-99a-dependent cell viability and apoptosis effect in breast cancer cells.

Activation of S6K1 protein enhances the translation of cellular mRNAs with a 5′-terminal oligopyrimidine tract and such mRNAs exclusively encode for components of the translation apparatus and control cell growth[35]–[37]. Activation of 4E-BP1 protein results in an increase in cap-dependent mRNAs (such as cyclin D1 and c-Myc), which also promote cell growth[38]. In contrast, inhibition of mTOR expression decreases expression of S6K1 and 4E-BP1, and subsequently leads to the reduction of mRNAs translation for negative regulators of cell cycle progression and cell proliferation[39]–[40], such as cyclin D1, c-Myc, Bcl-2, Bcl-xL and eIF4B[41]–[43]. Overexpression of 4E-BP1 or S6K1 leads to aggressive phenotypes of various cancer, such as advanced stages of disease and poor prognosis of breast cancer[44], [45]. In our current study, we found that the expression of phosphorylated 4E-BP1 and S6K1 was significantly reduced after inhibition of mTOR expression by miR-99a mimics in breast cell lines, concurrent with a reduction of cell viability and induction of apoptosis, while re-expression of mTOR could completely overcome the inhibitory effect of miR-99a on expression of mTOR/p-4E-BP1/p-S6K1 signal pathway genes. Thus, the current study provides a strong support of miR-99-targeted mTOR/p-4E-BP1/p-S6K1 signaling pathway in breast cancer cells. Further studies will explore whether targeting of this gene pathway could effectively treat breast cancer.

## Conclusions

Our study has further confirmed that miR-99a is a tumor suppressor gene, which is commonly downregulated in both breast cancer clinic tissues and breast cancer cell lines. Overexpression of miR-99a reduces breast cancer cell viability, induces apoptosis and inhibits tumorigenicity in vitro and in vivo through targeting mTOR/p-4E-BP1/p-S6K1 pathway. mTOR plays a critical role in mediating miR-99a dependent biological functions in breast cancer. miR-99a/mTOR might therefore be used as potential therapeutic targets in breast cancer.
